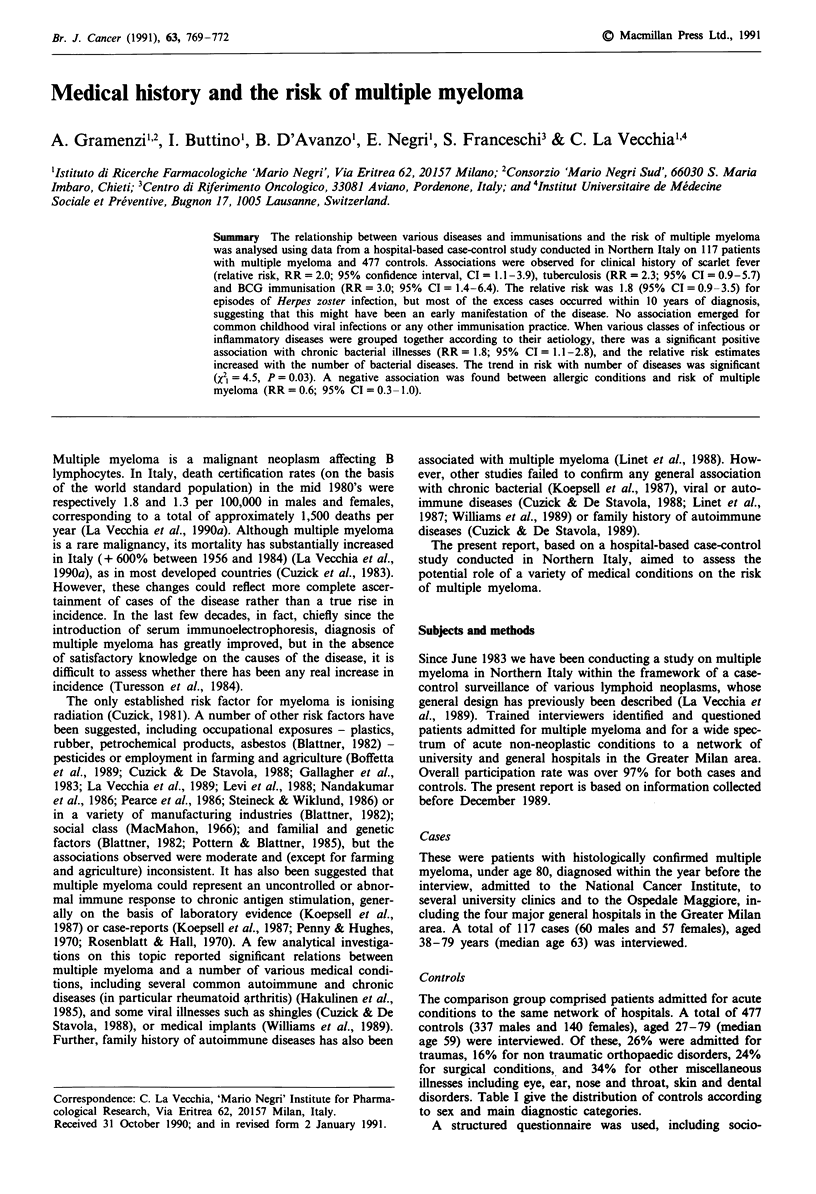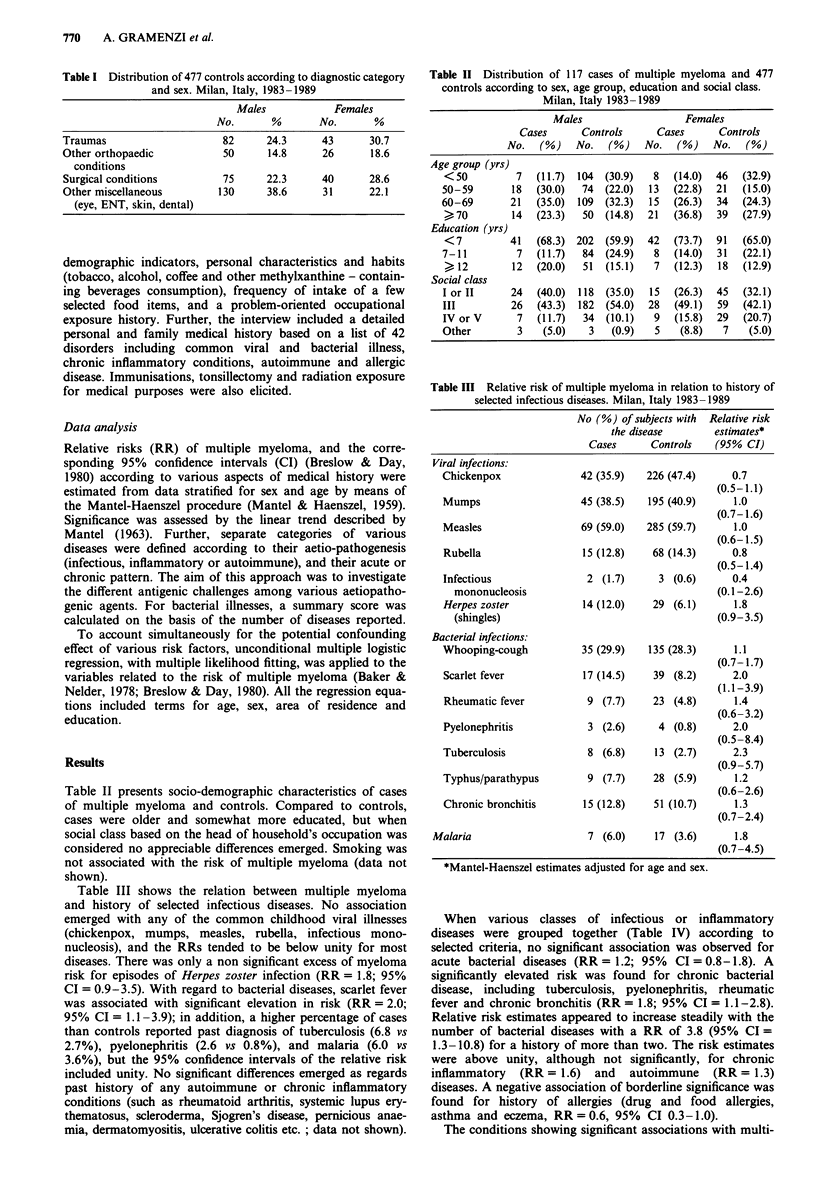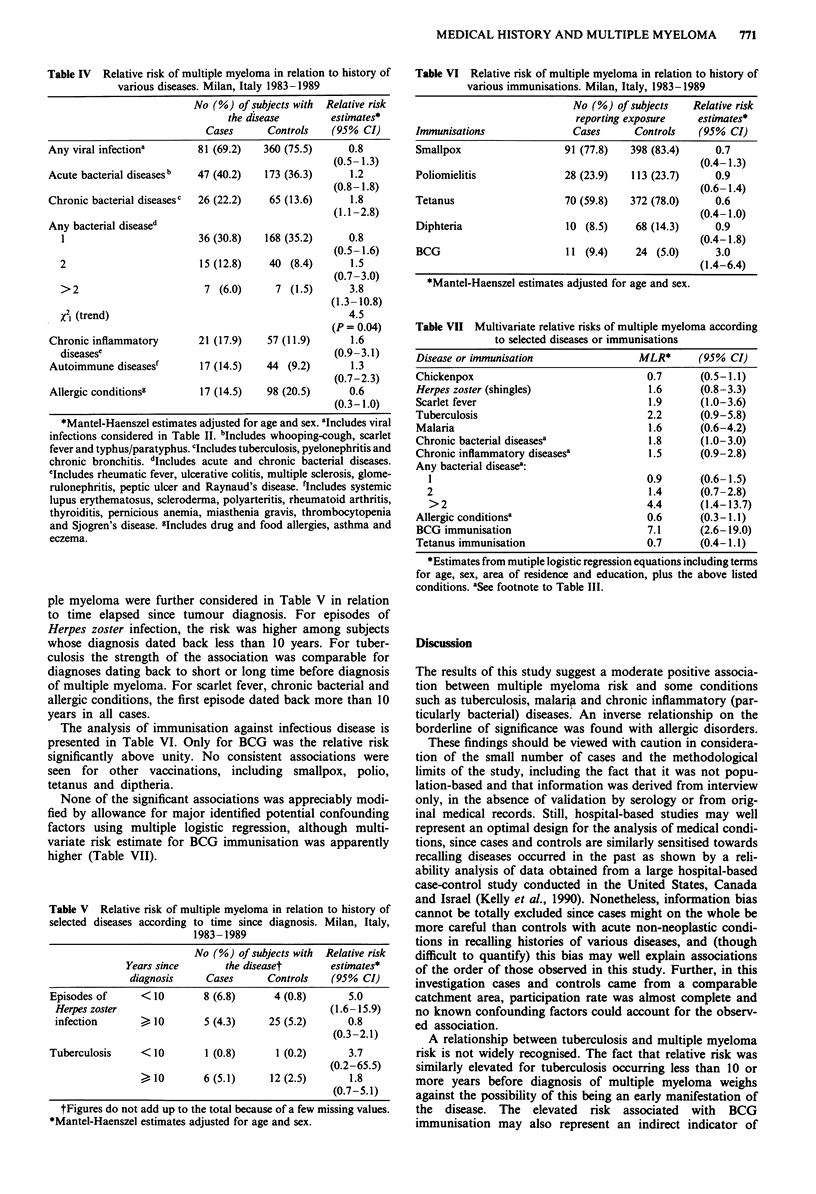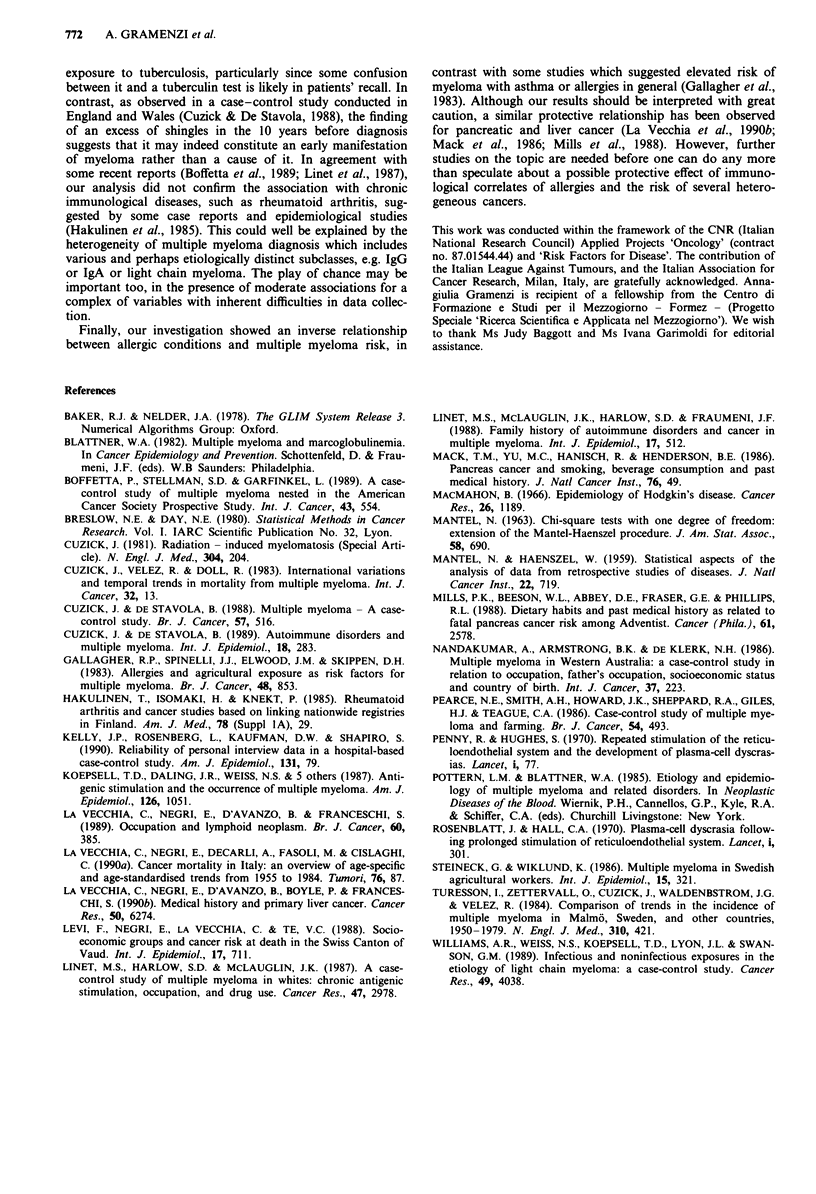# Medical history and the risk of multiple myeloma.

**DOI:** 10.1038/bjc.1991.172

**Published:** 1991-05

**Authors:** A. Gramenzi, I. Buttino, B. D'Avanzo, E. Negri, S. Franceschi, C. La Vecchia

**Affiliations:** Istituto di Ricerche Farmacologiche Mario Negri, Milano, Italy.

## Abstract

The relationship between various diseases and immunisations and the risk of multiple myeloma was analysed using data from a hospital-based case-control study conducted in Northern Italy on 117 patients with multiple myeloma and 477 controls. Associations were observed for clinical history of scarlet fever (relative risk, RR = 2.0; 95% confidence interval, CI = 1.1-3.9), tuberculosis (RR = 2.3%; 95% CI = 0.9-5.7) and BCG immunisation (RR = 3.0; 95% CI = 1.4-6.4). The relative risk was 1.8 (95% CI = 0.9-3.5) for episodes of Herpes zoster infection, but most of the excess cases occurred within 10 years of diagnosis, suggesting that this might have been an early manifestation of the disease. No association emerged for common childhood viral infections or any other immunisation practice. When various classes of infectious or inflammatory diseases were grouped together according to their aetiology, there was a significant positive association with chronic bacterial illnesses (RR = 1.8; 95% CI = 1.1-2.8), and the relative risk estimates increased with the number of bacterial diseases. The trend in risk with number of diseases was significant (chi 21 = 4.5, P = 0.03). A negative association was found between allergic conditions and risk of multiple myeloma (RR = 0.6; 95% CI = 0.3-1.0).


Br. J. Cancer (1991), 63, 769-772 © Macmillan Press Ltd., 1991

### Medical history and the risk of multiple myeloma

A. Gramenzi[1,2], I. Buttino[1], B. D'Avanzo[1], E. Negri[1], S. Franceschi[3] & C. La Vecchia[1,4]

[1]*Istituto di Ricerche Farmacologiche 'Mario Negri', Via Eritrea 62, 20157 Milano;* [2]*Consorzio 'Mario Negri Sud', 66030 S. Maria Imbaro, Chieti;* [3]*Centro di Riferimento Oncologico, 33081 Aviano, Pordenone, Italy; and* [4]*Institut Universitaire de Médecine Sociale et Préventive, Bugnon 17, 1005 Lausanne, Switzerland.*

**Summary** The relationship between various diseases and immunisations and the risk of multiple myeloma was analysed using data from a hospital-based case-control study conducted in Northern Italy on 117 patients with multiple myeloma and 477 controls. Associations were observed for clinical history of scarlet fever (relative risk, RR = 2.0; 95% confidence interval, CI = 1.1–3.9), tuberculosis (RR = 2.3; 95% CI = 0.9–5.7) and BCG immunisation (RR = 3.0; 95% CI = 1.4–6.4). The relative risk was 1.8 (95% CI = 0.9–3.5) for episodes of *Herpes zoster* infection, but most of the excess cases occurred within 10 years of diagnosis, suggesting that this might have been an early manifestation of the disease. No association emerged for common childhood viral infections or any other immunisation practice. When various classes of infectious or inflammatory diseases were grouped together according to their aetiology, there was a significant positive association with chronic bacterial illnesses (RR = 1.8; 95% CI = 1.1–2.8), and the relative risk estimates increased with the number of bacterial diseases. The trend in risk with number of diseases was significant ($\chi^2_1 = 4.5$, $P = 0.03$). A negative association was found between allergic conditions and risk of multiple myeloma (RR = 0.6; 95% CI = 0.3–1.0).

Multiple myeloma is a malignant neoplasm affecting B lymphocytes. In Italy, death certification rates (on the basis of the world standard population) in the mid 1980's were respectively 1.8 and 1.3 per 100,000 in males and females, corresponding to a total of approximately 1,500 deaths per year (La Vecchia *et al.*, 1990*a*). Although multiple myeloma is a rare malignancy, its mortality has substantially increased in Italy (+ 600% between 1956 and 1984) (La Vecchia *et al.*, 1990*a*), as in most developed countries (Cuzick *et al.*, 1983). However, these changes could reflect more complete ascertainment of cases of the disease rather than a true rise in incidence. In the last few decades, in fact, chiefly since the introduction of serum immunoelectrophoresis, diagnosis of multiple myeloma has greatly improved, but in the absence of satisfactory knowledge on the causes of the disease, it is difficult to assess whether there has been any real increase in incidence (Turesson *et al.*, 1984).

The only established risk factor for myeloma is ionising radiation (Cuzick, 1981). A number of other risk factors have been suggested, including occupational exposures – plastics, rubber, petrochemical products, asbestos (Blattner, 1982) – pesticides or employment in farming and agriculture (Boffetta *et al.*, 1989; Cuzick & De Stavola, 1988; Gallagher *et al.*, 1983; La Vecchia *et al.*, 1989; Levi *et al.*, 1988; Nandakumar *et al.*, 1986; Pearce *et al.*, 1986; Steineck & Wiklund, 1986) or in a variety of manufacturing industries (Blattner, 1982); social class (MacMahon, 1966); and familial and genetic factors (Blattner, 1982; Pottern & Blattner, 1985), but the associations observed were moderate and (except for farming and agriculture) inconsistent. It has also been suggested that multiple myeloma could represent an uncontrolled or abnormal immune response to chronic antigen stimulation, generally on the basis of laboratory evidence (Koepsell *et al.*, 1987) or case-reports (Koepsell *et al.*, 1987; Penny & Hughes, 1970; Rosenblatt & Hall, 1970). A few analytical investigations on this topic reported significant relations between multiple myeloma and a number of various medical conditions, including several common autoimmune and chronic diseases (in particular rheumatoid arthritis) (Hakulinen *et al.*, 1985), and some viral illnesses such as shingles (Cuzick & De Stavola, 1988), or medical implants (Williams *et al.*, 1989). Further, family history of autoimmune diseases has also been associated with multiple myeloma (Linet *et al.*, 1988). However, other studies failed to confirm any general association with chronic bacterial (Koepsell *et al.*, 1987), viral or autoimmune diseases (Cuzick & De Stavola, 1988; Linet *et al.*, 1987; Williams *et al.*, 1989) or family history of autoimmune diseases (Cuzick & De Stavola, 1989).

The present report, based on a hospital-based case-control study conducted in Northern Italy, aimed to assess the potential role of a variety of medical conditions on the risk of multiple myeloma.

### Subjects and methods

Since June 1983 we have been conducting a study on multiple myeloma in Northern Italy within the framework of a case-control surveillance of various lymphoid neoplasms, whose general design has previously been described (La Vecchia *et al.*, 1989). Trained interviewers identified and questioned patients admitted for multiple myeloma and for a wide spectrum of acute non-neoplastic conditions to a network of university and general hospitals in the Greater Milan area. Overall participation rate was over 97% for both cases and controls. The present report is based on information collected before December 1989.

### *Cases*

These were patients with histologically confirmed multiple myeloma, under age 80, diagnosed within the year before the interview, admitted to the National Cancer Institute, to several university clinics and to the Ospedale Maggiore, including the four major general hospitals in the Greater Milan area. A total of 117 cases (60 males and 57 females), aged 38–79 years (median age 63) was interviewed.

### *Controls*

The comparison group comprised patients admitted for acute conditions to the same network of hospitals. A total of 477 controls (337 males and 140 females), aged 27–79 (median age 59) were interviewed. Of these, 26% were admitted for traumas, 16% for non traumatic orthopaedic disorders, 24% for surgical conditions, and 34% for other miscellaneous illnesses including eye, ear, nose and throat, skin and dental disorders. Table I give the distribution of controls according to sex and main diagnostic categories.

A structured questionnaire was used, including socio-

Correspondence: C. La Vecchia, 'Mario Negri' Institute for Pharmacological Research, Via Eritrea 62, 20157 Milan, Italy.
Received 31 October 1990; and in revised form 2 January 1991.

**Table I** Distribution of 477 controls according to diagnostic category and sex. Milan, Italy, 1983–1989

| | *Males* | | *Females* | |
|---|---|---|---|---|
| | *No.* | *%* | *No.* | *%* |
| Traumas | 82 | 24.3 | 43 | 30.7 |
| Other orthopaedic conditions | 50 | 14.8 | 26 | 18.6 |
| Surgical conditions | 75 | 22.3 | 40 | 28.6 |
| Other miscellaneous (eye, ENT, skin, dental) | 130 | 38.6 | 31 | 22.1 |

demographic indicators, personal characteristics and habits (tobacco, alcohol, coffee and other methylxanthine – containing beverages consumption), frequency of intake of a few selected food items, and a problem-oriented occupational exposure history. Further, the interview included a detailed personal and family medical history based on a list of 42 disorders including common viral and bacterial illness, chronic inflammatory conditions, autoimmune and allergic disease. Immunisations, tonsillectomy and radiation exposure for medical purposes were also elicited.

### *Data analysis*

Relative risks (RR) of multiple myeloma, and the corresponding 95% confidence intervals (CI) (Breslow & Day, 1980) according to various aspects of medical history were estimated from data stratified for sex and age by means of the Mantel-Haenszel procedure (Mantel & Haenszel, 1959). Significance was assessed by the linear trend described by Mantel (1963). Further, separate categories of various diseases were defined according to their aetio-pathogenesis (infectious, inflammatory or autoimmune), and their acute or chronic pattern. The aim of this approach was to investigate the different antigenic challenges among various aetiopathogenic agents. For bacterial illnesses, a summary score was calculated on the basis of the number of diseases reported.

To account simultaneously for the potential confounding effect of various risk factors, unconditional multiple logistic regression, with multiple likelihood fitting, was applied to the variables related to the risk of multiple myeloma (Baker & Nelder, 1978; Breslow & Day, 1980). All the regression equations included terms for age, sex, area of residence and education.

### Results

Table II presents socio-demographic characteristics of cases of multiple myeloma and controls. Compared to controls, cases were older and somewhat more educated, but when social class based on the head of household's occupation was considered no appreciable differences emerged. Smoking was not associated with the risk of multiple myeloma (data not shown).

Table III shows the relation between multiple myeloma and history of selected infectious diseases. No association emerged with any of the common childhood viral illnesses (chickenpox, mumps, measles, rubella, infectious mononucleosis), and the RRs tended to be below unity for most diseases. There was only a non significant excess of myeloma risk for episodes of *Herpes zoster* infection (RR = 1.8; 95% CI = 0.9–3.5). With regard to bacterial diseases, scarlet fever was associated with significant elevation in risk (RR = 2.0; 95% CI = 1.1–3.9); in addition, a higher percentage of cases than controls reported past diagnosis of tuberculosis (6.8 *vs* 2.7%), pyelonephritis (2.6 *vs* 0.8%), and malaria (6.0 *vs* 3.6%), but the 95% confidence intervals of the relative risk included unity. No significant differences emerged as regards past history of any autoimmune or chronic inflammatory conditions (such as rheumatoid arthritis, systemic lupus erythematosus, scleroderma, Sjogren's disease, pernicious anaemia, dermatomyositis, ulcerative colitis etc. ; data not shown).

**Table II** Distribution of 117 cases of multiple myeloma and 477 controls according to sex, age group, education and social class. Milan, Italy 1983–1989

| | *Males* | | | | *Females* | | | |
|---|---|---|---|---|---|---|---|---|
| | *Cases* | | *Controls* | | *Cases* | | *Controls* | |
| | *No.* | *(%)* | *No.* | *(%)* | *No.* | *(%)* | *No.* | *(%)* |
| *Age group (yrs)* | | | | | | | | |
| <50 | 7 | (11.7) | 104 | (30.9) | 8 | (14.0) | 46 | (32.9) |
| 50–59 | 18 | (30.0) | 74 | (22.0) | 13 | (22.8) | 21 | (15.0) |
| 60–69 | 21 | (35.0) | 109 | (32.3) | 15 | (26.3) | 34 | (24.3) |
| ⩾70 | 14 | (23.3) | 50 | (14.8) | 21 | (36.8) | 39 | (27.9) |
| *Education (yrs)* | | | | | | | | |
| <7 | 41 | (68.3) | 202 | (59.9) | 42 | (73.7) | 91 | (65.0) |
| 7–11 | 7 | (11.7) | 84 | (24.9) | 8 | (14.0) | 31 | (22.1) |
| ⩾12 | 12 | (20.0) | 51 | (15.1) | 7 | (12.3) | 18 | (12.9) |
| *Social class* | | | | | | | | |
| I or II | 24 | (40.0) | 118 | (35.0) | 15 | (26.3) | 45 | (32.1) |
| III | 26 | (43.3) | 182 | (54.0) | 28 | (49.1) | 59 | (42.1) |
| IV or V | 7 | (11.7) | 34 | (10.1) | 9 | (15.8) | 29 | (20.7) |
| Other | 3 | (5.0) | 3 | (0.9) | 5 | (8.8) | 7 | (5.0) |

**Table III** Relative risk of multiple myeloma in relation to history of selected infectious diseases. Milan, Italy 1983–1989

| | *No (%) of subjects with the disease* | | *Relative risk estimates** |
|---|---|---|---|
| | *Cases* | *Controls* | *(95% CI)* |
| *Viral infections:* | | | |
| Chickenpox | 42 (35.9) | 226 (47.4) | 0.7 (0.5–1.1) |
| Mumps | 45 (38.5) | 195 (40.9) | 1.0 (0.7–1.6) |
| Measles | 69 (59.0) | 285 (59.7) | 1.0 (0.6–1.5) |
| Rubella | 15 (12.8) | 68 (14.3) | 0.8 (0.5–1.4) |
| Infectious mononucleosis | 2 (1.7) | 3 (0.6) | 0.4 (0.1–2.6) |
| *Herpes zoster* (shingles) | 14 (12.0) | 29 (6.1) | 1.8 (0.9–3.5) |
| *Bacterial infections:* | | | |
| Whooping-cough | 35 (29.9) | 135 (28.3) | 1.1 (0.7–1.7) |
| Scarlet fever | 17 (14.5) | 39 (8.2) | 2.0 (1.1–3.9) |
| Rheumatic fever | 9 (7.7) | 23 (4.8) | 1.4 (0.6–3.2) |
| Pyelonephritis | 3 (2.6) | 4 (0.8) | 2.0 (0.5–8.4) |
| Tuberculosis | 8 (6.8) | 13 (2.7) | 2.3 (0.9–5.7) |
| Typhus/parathypus | 9 (7.7) | 28 (5.9) | 1.2 (0.6–2.6) |
| Chronic bronchitis | 15 (12.8) | 51 (10.7) | 1.3 (0.7–2.4) |
| *Malaria* | 7 (6.0) | 17 (3.6) | 1.8 (0.7–4.5) |

*Mantel-Haenszel estimates adjusted for age and sex.

When various classes of infectious or inflammatory diseases were grouped together (Table IV) according to selected criteria, no significant association was observed for acute bacterial diseases (RR = 1.2; 95% CI = 0.8–1.8). A significantly elevated risk was found for chronic bacterial disease, including tuberculosis, pyelonephritis, rheumatic fever and chronic bronchitis (RR = 1.8; 95% CI = 1.1–2.8). Relative risk estimates appeared to increase steadily with the number of bacterial diseases with a RR of 3.8 (95% CI = 1.3–10.8) for a history of more than two. The risk estimates were above unity, although not significantly, for chronic inflammatory (RR = 1.6) and autoimmune (RR = 1.3) diseases. A negative association of borderline significance was found for history of allergies (drug and food allergies, asthma and eczema, RR = 0.6, 95% CI 0.3–1.0).

The conditions showing significant associations with multi-

**Table IV** Relative risk of multiple myeloma in relation to history of various diseases. Milan, Italy 1983–1989

| | *No (%) of subjects with the disease* | | *Relative risk estimates** |
|---|---|---|---|
| | *Cases* | *Controls* | *(95% CI)* |
| Any viral infection[a] | 81 (69.2) | 360 (75.5) | 0.8 (0.5–1.3) |
| Acute bacterial diseases[b] | 47 (40.2) | 173 (36.3) | 1.2 (0.8–1.8) |
| Chronic bacterial diseases[c] | 26 (22.2) | 65 (13.6) | 1.8 (1.1–2.8) |
| Any bacterial disease[d] | | | |
| 1 | 36 (30.8) | 168 (35.2) | 0.8 (0.5–1.6) |
| 2 | 15 (12.8) | 40 (8.4) | 1.5 (0.7–3.0) |
| >2 | 7 (6.0) | 7 (1.5) | 3.8 (1.3–10.8) |
| $\chi^2_1$ (trend) | | | 4.5 ($P$ = 0.04) |
| Chronic inflammatory diseases[e] | 21 (17.9) | 57 (11.9) | 1.6 (0.9–3.1) |
| Autoimmune diseases[f] | 17 (14.5) | 44 (9.2) | 1.3 (0.7–2.3) |
| Allergic conditions[g] | 17 (14.5) | 98 (20.5) | 0.6 (0.3–1.0) |

*Mantel-Haenszel estimates adjusted for age and sex. [a]Includes viral infections considered in Table II. [b]Includes whooping-cough, scarlet fever and typhus/paratyphus. [c]Includes tuberculosis, pyelonephritis and chronic bronchitis. [d]Includes acute and chronic bacterial diseases. [e]Includes rheumatic fever, ulcerative colitis, multiple sclerosis, glomerulonephritis, peptic ulcer and Raynaud's disease. [f]Includes systemic lupus erythematosus, scleroderma, polyarteritis, rheumatoid arthritis, thyroiditis, pernicious anemia, miasthenia gravis, thrombocytopenia and Sjogren's disease. [g]Includes drug and food allergies, asthma and eczema.

ple myeloma were further considered in Table V in relation to time elapsed since tumour diagnosis. For episodes of *Herpes zoster* infection, the risk was higher among subjects whose diagnosis dated back less than 10 years. For tuberculosis the strength of the association was comparable for diagnoses dating back to short or long time before diagnosis of multiple myeloma. For scarlet fever, chronic bacterial and allergic conditions, the first episode dated back more than 10 years in all cases.

The analysis of immunisation against infectious disease is presented in Table VI. Only for BCG was the relative risk significantly above unity. No consistent associations were seen for other vaccinations, including smallpox, polio, tetanus and diptheria.

None of the significant associations was appreciably modified by allowance for major identified potential confounding factors using multiple logistic regression, although multivariate risk estimate for BCG immunisation was apparently higher (Table VII).

**Table V** Relative risk of multiple myeloma in relation to history of selected diseases according to time since diagnosis. Milan, Italy, 1983–1989

| | *Years since diagnosis* | *No (%) of subjects with the disease†* | | *Relative risk estimates** |
|---|---|---|---|---|
| | | *Cases* | *Controls* | *(95% CI)* |
| Episodes of *Herpes zoster* infection | <10 | 8 (6.8) | 4 (0.8) | 5.0 (1.6–15.9) |
| | ⩾10 | 5 (4.3) | 25 (5.2) | 0.8 (0.3–2.1) |
| Tuberculosis | <10 | 1 (0.8) | 1 (0.2) | 3.7 (0.2–65.5) |
| | ⩾10 | 6 (5.1) | 12 (2.5) | 1.8 (0.7–5.1) |

†Figures do not add up to the total because of a few missing values. *Mantel-Haenszel estimates adjusted for age and sex.

**Table VI** Relative risk of multiple myeloma in relation to history of various immunisations. Milan, Italy, 1983–1989

| *Immunisations* | *No (%) of subjects reporting exposure* | | *Relative risk estimates** |
|---|---|---|---|
| | *Cases* | *Controls* | *(95% CI)* |
| Smallpox | 91 (77.8) | 398 (83.4) | 0.7 (0.4–1.3) |
| Poliomielitis | 28 (23.9) | 113 (23.7) | 0.9 (0.6–1.4) |
| Tetanus | 70 (59.8) | 372 (78.0) | 0.6 (0.4–1.0) |
| Diphteria | 10 (8.5) | 68 (14.3) | 0.9 (0.4–1.8) |
| BCG | 11 (9.4) | 24 (5.0) | 3.0 (1.4–6.4) |

*Mantel-Haenszel estimates adjusted for age and sex.

**Table VII** Multivariate relative risks of multiple myeloma according to selected diseases or immunisations

| *Disease or immunisation* | *MLR** | *(95% CI)* |
|---|---|---|
| Chickenpox | 0.7 | (0.5–1.1) |
| *Herpes zoster* (shingles) | 1.6 | (0.8–3.3) |
| Scarlet fever | 1.9 | (1.0–3.6) |
| Tuberculosis | 2.2 | (0.9–5.8) |
| Malaria | 1.6 | (0.6–4.2) |
| Chronic bacterial diseases[a] | 1.8 | (1.0–3.0) |
| Chronic inflammatory diseases[a] | 1.5 | (0.9–2.8) |
| Any bacterial disease[a]: | | |
| 1 | 0.9 | (0.6–1.5) |
| 2 | 1.4 | (0.7–2.8) |
| >2 | 4.4 | (1.4–13.7) |
| Allergic conditions[a] | 0.6 | (0.3–1.1) |
| BCG immunisation | 7.1 | (2.6–19.0) |
| Tetanus immunisation | 0.7 | (0.4–1.1) |

*Estimates from mutiple logistic regression equations including terms for age, sex, area of residence and education, plus the above listed conditions. [a]See footnote to Table III.

### Discussion

The results of this study suggest a moderate positive association between multiple myeloma risk and some conditions such as tuberculosis, malaria and chronic inflammatory (particularly bacterial) diseases. An inverse relationship on the borderline of significance was found with allergic disorders.

These findings should be viewed with caution in consideration of the small number of cases and the methodological limits of the study, including the fact that it was not population-based and that information was derived from interview only, in the absence of validation by serology or from original medical records. Still, hospital-based studies may well represent an optimal design for the analysis of medical conditions, since cases and controls are similarly sensitised towards recalling diseases occurred in the past as shown by a reliability analysis of data obtained from a large hospital-based case-control study conducted in the United States, Canada and Israel (Kelly *et al.*, 1990). Nonetheless, information bias cannot be totally excluded since cases might on the whole be more careful than controls with acute non-neoplastic conditions in recalling histories of various diseases, and (though difficult to quantify) this bias may well explain associations of the order of those observed in this study. Further, in this investigation cases and controls came from a comparable catchment area, participation rate was almost complete and no known confounding factors could account for the observed association.

A relationship between tuberculosis and multiple myeloma risk is not widely recognised. The fact that relative risk was similarly elevated for tuberculosis occurring less than 10 or more years before diagnosis of multiple myeloma weighs against the possibility of this being an early manifestation of the disease. The elevated risk associated with BCG immunisation may also represent an indirect indicator of exposure to tuberculosis, particularly since some confusion between it and a tuberculin test is likely in patients' recall. In contrast, as observed in a case–control study conducted in England and Wales (Cuzick & De Stavola, 1988), the finding of an excess of shingles in the 10 years before diagnosis suggests that it may indeed constitute an early manifestation of myeloma rather than a cause of it. In agreement with some recent reports (Boffetta *et al.*, 1989; Linet *et al.*, 1987), our analysis did not confirm the association with chronic immunological diseases, such as rheumatoid arthritis, suggested by some case reports and epidemiological studies (Hakulinen *et al.*, 1985). This could well be explained by the heterogeneity of multiple myeloma diagnosis which includes various and perhaps etiologically distinct subclasses, e.g. IgG or IgA or light chain myeloma. The play of chance may be important too, in the presence of moderate associations for a complex of variables with inherent difficulties in data collection.

Finally, our investigation showed an inverse relationship between allergic conditions and multiple myeloma risk, in contrast with some studies which suggested elevated risk of myeloma with asthma or allergies in general (Gallagher *et al.*, 1983). Although our results should be interpreted with great caution, a similar protective relationship has been observed for pancreatic and liver cancer (La Vecchia *et al.*, 1990*b*; Mack *et al.*, 1986; Mills *et al.*, 1988). However, further studies on the topic are needed before one can do any more than speculate about a possible protective effect of immunological correlates of allergies and the risk of several heterogeneous cancers.

This work was conducted within the framework of the CNR (Italian National Research Council) Applied Projects 'Oncology' (contract no. 87.01544.44) and 'Risk Factors for Disease'. The contribution of the Italian League Against Tumours, and the Italian Association for Cancer Research, Milan, Italy, are gratefully acknowledged. Annagiulia Gramenzi is recipient of a fellowship from the Centro di Formazione e Studi per il Mezzogiorno – Formez – (Progetto Speciale 'Ricerca Scientifica e Applicata nel Mezzogiorno'). We wish to thank Ms Judy Baggott and Ms Ivana Garimoldi for editorial assistance.

### References

BAKER, R.J. & NELDER, J.A. (1978). *The GLIM System Release 3.* Numerical Algorithms Group: Oxford.

BLATTNER, W.A. (1982). Multiple myeloma and marcoglobulinemia. In *Cancer Epidemiology and Prevention.* Schottenfeld, D. & Fraumeni, J.F. (eds). W.B Saunders: Philadelphia.

BOFFETTA, P., STELLMAN, S.D. & GARFINKEL, L. (1989). A case-control study of multiple myeloma nested in the American Cancer Society Prospective Study. *Int. J. Cancer*, **43**, 554.

BRESLOW, N.E. & DAY, N.E. (1980). *Statistical Methods in Cancer Research.* Vol. I. IARC Scientific Publication No. 32, Lyon.

CUZICK, J. (1981). Radiation – induced myelomatosis (Special Article). *N. Engl. J. Med.*, **304**, 204.

CUZICK, J., VELEZ, R. & DOLL, R. (1983). International variations and temporal trends in mortality from multiple myeloma. *Int. J. Cancer*, **32**, 13.

CUZICK, J. & DE STAVOLA, B. (1988). Multiple myeloma – A case-control study. *Br. J. Cancer*, **57**, 516.

CUZICK, J. & DE STAVOLA, B. (1989). Autoimmune disorders and multiple myeloma. *Int. J. Epidemiol.*, **18**, 283.

GALLAGHER, R.P., SPINELLI, J.J., ELWOOD, J.M. & SKIPPEN, D.H. (1983). Allergies and agricultural exposure as risk factors for multiple myeloma. *Br. J. Cancer*, **48**, 853.

HAKULINEN, T., ISOMAKI, H. & KNEKT, P. (1985). Rheumatoid arthritis and cancer studies based on linking nationwide registries in Finland. *Am. J. Med.*, **78** (Suppl 1A), 29.

KELLY, J.P., ROSENBERG, L., KAUFMAN, D.W. & SHAPIRO, S. (1990). Reliability of personal interview data in a hospital-based case-control study. *Am. J. Epidemiol.*, **131**, 79.

KOEPSELL, T.D., DALING, J.R., WEISS, N.S. & 5 others (1987). Antigenic stimulation and the occurrence of multiple myeloma. *Am. J. Epidemiol.*, **126**, 1051.

LA VECCHIA, C., NEGRI, E., D'AVANZO, B. & FRANCESCHI, S. (1989). Occupation and lymphoid neoplasm. *Br. J. Cancer*, **60**, 385.

LA VECCHIA, C., NEGRI, E., DECARLI, A., FASOLI, M. & CISLAGHI, C. (1990*a*). Cancer mortality in Italy: an overview of age-specific and age-standardised trends from 1955 to 1984. *Tumori*, **76**, 87.

LA VECCHIA, C., NEGRI, E., D'AVANZO, B., BOYLE, P. & FRANCESCHI, S. (1990*b*). Medical history and primary liver cancer. *Cancer Res.*, **50**, 6274.

LEVI, F., NEGRI, E., LA VECCHIA, C. & TE, V.C. (1988). Socioeconomic groups and cancer risk at death in the Swiss Canton of Vaud. *Int. J. Epidemiol.*, **17**, 711.

LINET, M.S., HARLOW, S.D. & MCLAUGLIN, J.K. (1987). A case-control study of multiple myeloma in whites: chronic antigenic stimulation, occupation, and drug use. *Cancer Res.*, **47**, 2978.

LINET, M.S., MCLAUGLIN, J.K., HARLOW, S.D. & FRAUMENI, J.F. (1988). Family history of autoimmune disorders and cancer in multiple myeloma. *Int. J. Epidemiol.*, **17**, 512.

MACK, T.M., YU, M.C., HANISCH, R. & HENDERSON, B.E. (1986). Pancreas cancer and smoking, beverage consumption and past medical history. *J. Natl Cancer Inst.*, **76**, 49.

MACMAHON, B. (1966). Epidemiology of Hodgkin's disease. *Cancer Res.*, **26**, 1189.

MANTEL, N. (1963). Chi-square tests with one degree of freedom: extension of the Mantel-Haenszel procedure. *J. Am. Stat. Assoc.*, **58**, 690.

MANTEL, N. & HAENSZEL, W. (1959). Statistical aspects of the analysis of data from retrospective studies of diseases. *J. Natl Cancer Inst.*, **22**, 719.

MILLS, P.K., BEESON, W.L., ABBEY, D.E., FRASER, G.E. & PHILLIPS, R.L. (1988). Dietary habits and past medical history as related to fatal pancreas cancer risk among Adventist. *Cancer (Phila.)*, **61**, 2578.

NANDAKUMAR, A., ARMSTRONG, B.K. & DE KLERK, N.H. (1986). Multiple myeloma in Western Australia: a case-control study in relation to occupation, father's occupation, socioeconomic status and country of birth. *Int. J. Cancer*, **37**, 223.

PEARCE, N.E., SMITH, A.H., HOWARD, J.K., SHEPPARD, R.A., GILES, H.J. & TEAGUE, C.A. (1986). Case-control study of multiple myeloma and farming. *Br. J. Cancer*, **54**, 493.

PENNY, R. & HUGHES, S. (1970). Repeated stimulation of the reticuloendothelial system and the development of plasma-cell dyscrasias. *Lancet*, **i**, 77.

POTTERN, L.M. & BLATTNER, W.A. (1985). Etiology and epidemiology of multiple myeloma and related disorders. In *Neoplastic Diseases of the Blood.* Wiernik, P.H., Cannellos, G.P., Kyle, R.A. & Schiffer, C.A. (eds). Churchill Livingstone: New York.

ROSENBLATT, J. & HALL, C.A. (1970). Plasma-cell dyscrasia following prolonged stimulation of reticuloendothelial system. *Lancet*, **i**, 301.

STEINECK, G. & WIKLUND, K. (1986). Multiple myeloma in Swedish agricultural workers. *Int. J. Epidemiol.*, **15**, 321.

TURESSON, I., ZETTERVALL, O., CUZICK, J., WALDENBSTROM, J.G. & VELEZ, R. (1984). Comparison of trends in the incidence of multiple myeloma in Malmö, Sweden, and other countries, 1950–1979. *N. Engl. J. Med.*, **310**, 421.

WILLIAMS, A.R., WEISS, N.S., KOEPSELL, T.D., LYON, J.L. & SWANSON, G.M. (1989). Infectious and noninfectious exposures in the etiology of light chain myeloma: a case-control study. *Cancer Res.*, **49**, 4038.